# Decreased Neuron Density and Increased Glia Density in the Ventromedial Prefrontal Cortex (Brodmann Area 25) in Williams Syndrome

**DOI:** 10.3390/brainsci8120209

**Published:** 2018-11-29

**Authors:** Linnea Wilder, Kari L. Hanson, Caroline H. Lew, Ursula Bellugi, Katerina Semendeferi

**Affiliations:** 1 Department of Anthropology, Social Sciences Building Rm. 210, University of California, San Diego, 9500 Gilman Drive, La Jolla, CA 92093-0532, USA; llwilder@ucsd.edu (L.W.); k1hanson@ucsd.edu (K.L.H.); cfhorton@ucsd.edu (C.H.L.); 2 Laboratory for Cognitive Neuroscience, Salk Institute for Biological Studies, 10010 N. Torrey Pines Rd., La Jolla, CA 92037, USA; bellugi@salk.edu

**Keywords:** Williams Syndrome, neuron density, glia density, ventromedial prefrontal cortex

## Abstract

Williams Syndrome (WS) is a neurodevelopmental disorder caused by a deletion of 25–28 genes on chromosome 7 and characterized by a specific behavioral phenotype, which includes hypersociability and anxiety. Here, we examined the density of neurons and glia in fourteen human brains in Brodmann area 25 (BA 25), in the ventromedial prefrontal cortex (vmPFC), using a postmortem sample of five adult and two infant WS brains and seven age-, sex- and hemisphere-matched typically developing control (TD) brains. We found decreased neuron density, which reached statistical significance in the supragranular layers, and increased glia density and glia to neuron ratio, which reached statistical significance in both supra- and infragranular layers. Combined with our previous findings in the amygdala, caudate nucleus and frontal pole (BA 10), these results in the vmPFC suggest that abnormalities in frontostriatal and frontoamygdala circuitry may contribute to the anxiety and atypical social behavior observed in WS.

## 1. Introduction

Williams Syndrome (WS) is a rare (<1 in 7500) neurodevelopmental disorder resulting from a deletion of approximately 25–28 genes on chromosome band 7q11.23 [1]. Individuals with WS have a specific and well defined cognitive and behavioral phenotype. The cognitive profile of WS is characterized by deficits in global IQ and spatial processing, and relatively preserved language and face processing. However, even in these relatively spared skills, WS individuals demonstrate delayed and abnormal development, along with atypical cognitive processing during some language and face tasks [2,3]. WS behavior is marked by high levels of sociability and anxiety. WS individuals have a high drive to engage in social interactions with others, and a tendency to approach even unfamiliar individuals to engage them in conversation [4,5]. In striking contrast to this, Autism Spectrum Disorders (ASD) are characterized often by social avoidance [6,7]. Unlike WS, ASD are genetically complex and heterogenous [6,7]. Interestingly, however, duplication of the WS gene deletion appears to cause ASD in a small subset of cases, demonstrating the range of behavioral effects that alterations at this locus can cause [8].

Abnormalities in the structure and function of the prefrontal cortex (PFC) have been demonstrated in imaging studies of WS. Overall cortical surface area, including surface area in two regions linked to emotion processing and social behavior, the orbital and medial prefrontal cortices, is decreased in WS. Cortical thickness, however, appears increased in these regions, and relative to brain size, total gray matter volume may also be increased in the orbital and medial prefrontal cortices in WS [9,10,11]. Functional imaging studies (fMRI) provide evidence of deficits in behavioral inhibition in WS. WS subjects had slower response times on an inhibition task than TD controls and displayed lower levels of activation in the striatum and frontal cortex [12]. These abnormalities in fronto-striatal circuitry, and deficits in behavioral inhibition may relate to WS hypersociability, which has been described as an inability to inhibit the desire to approach and engage with others [13,14]. In a functional imaging study examining response to threat, WS individuals displayed lower levels of activation in the amygdala and ventromedial prefrontal cortex vmPFC while viewing threatening faces, but higher levels of activation in these regions while viewing threatening scenes, compared to TD controls [15]. Atypical communication between frontal and limbic regions has been suggested as a possible factor in the high anxiety seen in WS [16]. At the cellular level, microstructural analyses of WS subjects demonstrated lower neuronal density in the infragranular layers of the rostral orbitofrontal cortex [17]. An increase in the ratio of glia to neurons, and in the density of oligodendrocytes in WS, has been found in the in the medial caudate nucleus, a region that receives projections from the vmPFC [18,19]. In the amygdala of WS subjects, neuron number was higher in the lateral nucleus [20]. Taken together, these findings suggest that abnormalities in PFC cytoarchitecture, and altered prefrontal inhibitory control of the amygdala and striatum, may be linked to the atypical anxiety and social behavior characteristic of WS.

Here, we examined one area of the vmPFC, Brodmann area 25 (BA 25), that is critically involved in social behaviors and related functions of inhibition and decision making [21]. This area is heavily connected to several subcortical structures, including the amygdala and striatum, both of which are altered in WS, and in other disorders including autism [18,20,22,23,24]. Using postmortem tissue from ten adult and four infant subjects, seven WS and seven age, sex, and hemisphere matched typically developing (TD) controls, we measured the density of neurons and glia in the supragranular (II/III) and infragranular (V/VI) layers of BA 25 in the vmPFC to test whether the previously observed decreases in neuron density in WS are restricted to rostral orbitofrontal cortical areas, or if there are widespread alterations to the frontal cortex in WS.

## 2. Materials and Methods

### 2.1. Brain Tissue

We examined cortical tissue from BA 25 in the vmPFC in a total of fourteen postmortem human subjects, including five adult WS and five adult TD subjects, as well as two WS infant subjects and two TD infant subjects (Table 1). TD subjects were matched with WS subjects for age (110/114 and 234/245 days for infants, 18–43 years for adults), sex, and hemisphere (right), to control for possible cytoarchitectonic asymmetries and age and sex-related differences [25,26].

All subjects in the Bellugi Williams Syndrome Brain Collection are part of an ongoing donation-based program now run by the Laboratory for Human Comparative Neuroanatomy at UCSD (La Jolla, CA, USA).

### 2.2. Regions of Interest

**Figure 1:**
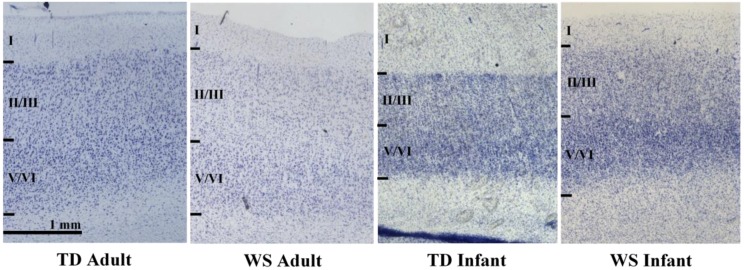
Microphotographs of Brodmann area 25 (BA 25) in adult and infant Williams Syndrome (WS) and typically developing control (TD). Images taken at 2×.

The region of interest (ROI) was identified using anatomical landmarks and by the absence of any visible border between cortical layers II and III and between layers V and VI. BA 25 occupies a portion of the brain immediately caudal and ventral to genu of the corpus callosum. It is agranular, lacking a visible layer IV, and poorly laminated compared to surrounding cortical areas [27,28]. Cortical layers II/III and V/VI were analyzed as two distinct ROIs (Figure 1).

### 2.3. Processing of Tissue

Blocks of tissue containing BA 25 were extracted and cryoprotected using a series of 10%, 20%, and 30% sucrose solutions with 0.1 M phosphate buffer until saturated. Frozen tissue was cut on a Leica SM 2010R (Leica Biosystems, Wetzlar, Germany) sliding microtome into ten series of 40 micrometer (µm) thick sections in adult subjects. Due to the fragility of infant tissue, infant subjects were cut into five series of 80 µm thick sections. One series was rehydrated for 48 h in a neutral phosphate buffer, then mounted on gelatin-coated slides. Mounted sections were dried for 48 h at room temperature, then dehydrated in a 1:1 chloroform ethanol solution overnight. These sections were stained with a 0.25% thionine stain for Nissl substance to visualize cell bodies, rehydrated, submerged in xylenes or citrisolv for 15 min after staining, and then cover-slipped with permount. The remaining series were stored for use in later processing, including a variety of immunohistological staining experiments.

### 2.4. Unbiased, Design-Based Stereology

Data collection was performed using StereoInvestigator software (MBF Bioscience, Williston, VT, USA) on a Dell workstation receiving live video feed from an Optronics MicroFire color video camera (East Muskogee, OK, USA) attached to a Nikon Eclipse 80i microscope (Nikon Instruments, Melville, NY, USA) equipped with a Ludl MAC5000 stage (Ludl, Hawthorn, NY, USA) and a Heidenhain z-axis encoder (Heidenhain, Plymouth, MN, USA). To increase the accuracy and consistency of measurements across all subjects, we report neuron and glia density rather than number, a standard practice for data collection in the cortex [17,29,30,31].

**Figure 2:**
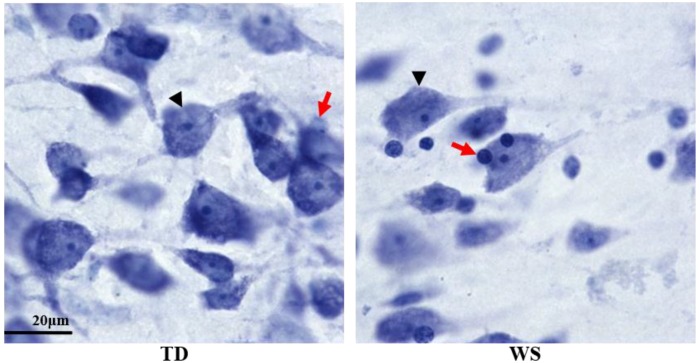
Microphotograph of BA 25 adult WS and TD. Neurons (black arrowheads) were distinguished from glia (red arrows) by their large size and distinctly stained nucleolus. Images taken at 100×.

All data were collected by a single rater (LW). Inter-rater reliability was ensured through repeated neuron density estimations on a sample previously reported in the literature to 95% concordance [17]. Sections were coded before data collection to blind the rater to diagnosis. Six sections per subject, spaced as equidistantly as allowed by individual section quality, were analyzed, representing the maximum extent of the area in the coronal plane. Neuron and glia densities in layers II/III and V/VI were estimated using the Optical Fractionator probe in StereoInvestigator. Two regions of interest per section, one bounding layers II/III and the other bounding layers V/VI, were drawn at a 1× magnification, consistent with previous work on WS cortex [17]. BA 25 in adults has no visible layer IV. Neurons and glia were counted using a 1.4 numerical aperture, 100× oil objective lens, with a grid size of 300 × 300 microns, a dissector height of 9 microns, and a counting frame of 85 × 85 microns. For infant subjects, a 50 × 50 micron counting frame was used. Within this frame, neurons and glia not touching the line of exclusion were counted using different markers. Cells were distinguished based on their morphology. Neurons were identified by the presence of a distinct nucleolus, and a lightly stained nucleus surrounded by cytoplasm. Glia were identified by their smaller size and lightly or darkly stained nucleus, with very little or no staining of the surrounding cytoplasm (Figure 2) [32]. For each ROI (layers II/III and layers V/VI, respectively), neuron and glia densities were calculated by dividing population estimate of each cell type by the planimetric volume estimate from the Optical Fractionator probe.

### 2.5. Statistical Analysis

Standard two-tailed *t*-tests (*p* < 0.05) were used to compare neuron density, glia density, and glia to neuron ratio in WS and TD. Supragranular and infragranular layers were compared separately, as well as the average density of these layers combined. Percent difference in WS compared to TD was calculated as the difference in mean value of WS from TD, in relation to the mean TD value, for neuron density, glia density, and glia to neuron ratio, in each ROI.

## 3. Results

### 3.1. Adult Neuron Density

**Figure 3:**
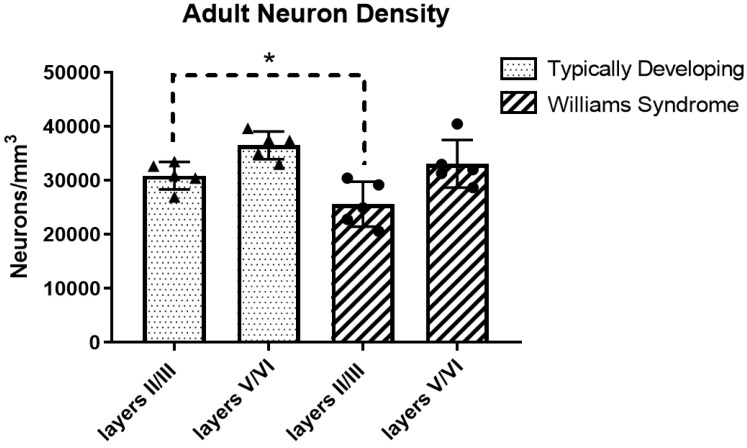
Mean neuron density in adults. * Statistically significant results.

Results are summarized in Table 2 and Figure 3. In supragranular layers, neuron density was significantly decreased in WS compared to TD (*p* = 0.046, 17% decrease). Neuron density infragranular layers were decreased in WS, but this was not statistically significant (*p* = 0.186, 9% decrease).

### 3.2. Adult Glia Density and Glia to Neuron Ratio

**Figure 4:**
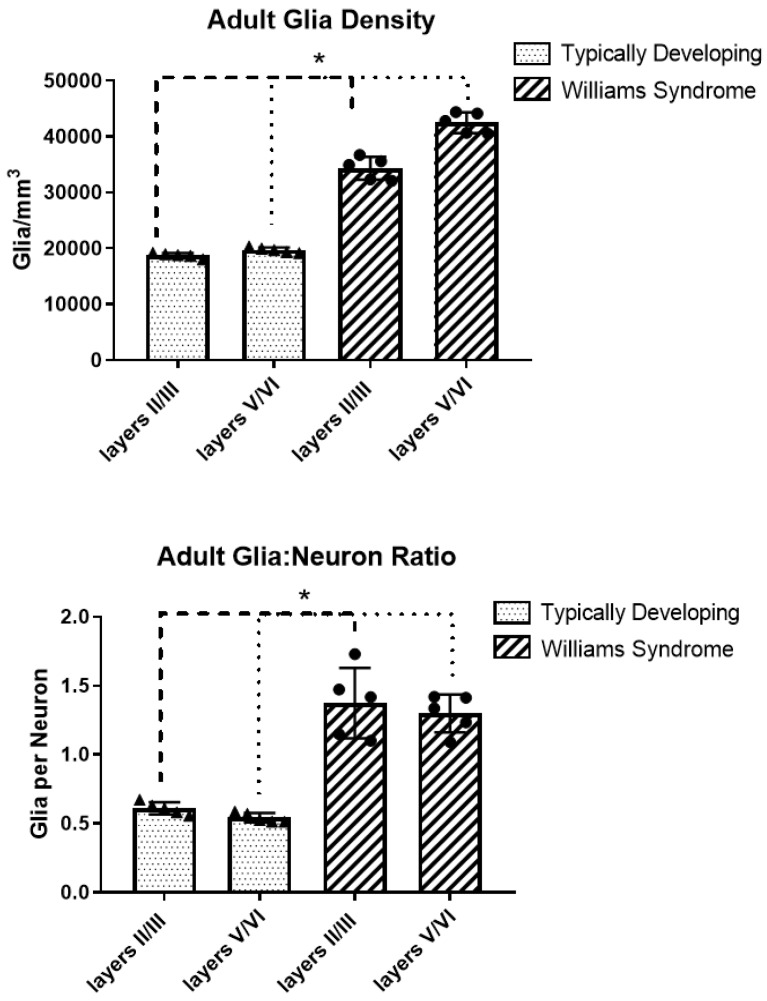
Mean glia density and glia to neuron ratio in adults. * Statistically significant results.

Results are summarized in Table 3 and Table 4, and Figure 4. Mean glia density was significantly increased in WS compared to TD, in both supragranular (83% increase, *p* = 0.00007) and infragranular (116% increase, *p* = 0.000001) layers. Glia to neuron ratio was also increased in WS compared to TD in supragranular (125% increase, *p* = 0.003) and infragranular (140%, *p* = 0.0003) layers.

### 3.3. Infant Neuron Density, Glia Density, and Glia to Neuron Ratio

**Figure 5:**
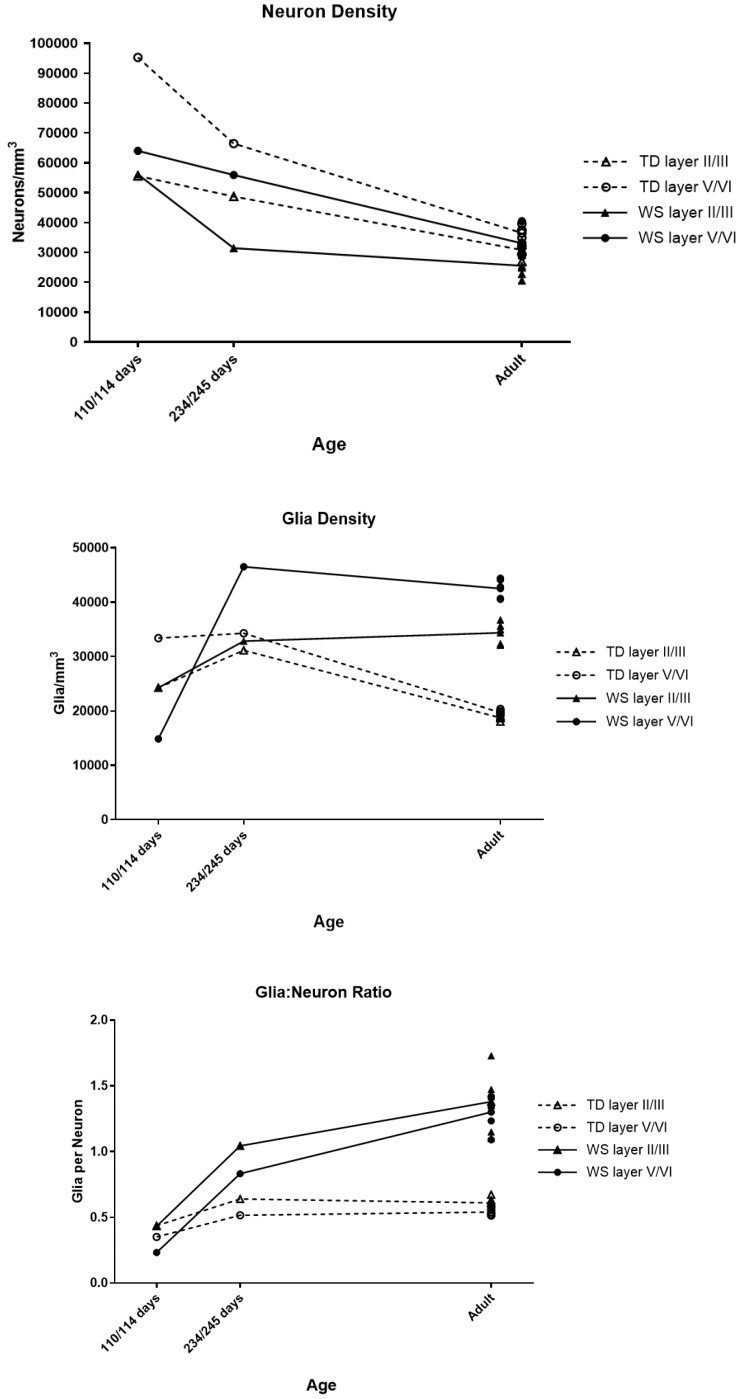
Neuron density, glia density, and glia to neuron ratio in infants.

Results are summarized in Figure 5. In the 114 (WS) and 110 (TD) day-old subject pair, neuron density, glia density, and glia to neuron ratio were quite similar between the TD and WS subject in the supragranular layers (within 1%). In the infragranular layers, neuron density, glia density, and glia to neuron ratio were lower in the WS subject (33% lower, 55% lower, and 16% lower respectively). In the 234- and 245-day pair, across all layers, neuron density was lower (35% lower supragranular, 16% lower infragranular), and glia density (5% higher supragranular, 16% higher infragranular) and glia to neuron ratio (63% higher supragranular, 61% higher infragranular) were both higher in the WS subject.

Table 5 summarizes results for all ages.

## 4. Discussion

Very few histological studies of BA 25 in TD adults have been conducted [27,33,34], and none on infants. All data from these few adult studies are qualitative rather than quantitative. The present study provides the first quantitative data for neuron and glia density in TD adult and infant BA 25. Our findings demonstrate variation in cell density between cortical layers consistent with expected patterns based on adult TD brains from the limited reports available. Qualitative description of human BA 25, along with quantitative findings in macaques, show that caudal, agranular regions of the vmPFC, such as BA 25, are characterized by higher neuron density in infragranular layers compared to supragranular layers, and lower glia density than neuron density in all layers [27,28]. As expected, neuron density is much higher in infants than adults [35]. This study builds on previous research on the adult PFC in both TD and WS, a region implicated in the behavioral phenotype of the disorder, and is the first to examine the PFC in WS infants [9,17,35].

Adults: A previous postmortem histological study of WS, which included three of the same adult WS subjects utilized here, along with an additional three adult WS subjects and six adult TD controls, found decreased neuron density in BA 10 and 11 of the prefrontal cortex (PFC), with the greatest difference observed in the infragranular layers [17]. A study on the morphology of basal dendrites in adult WS subjects found that dendritic length and branching were compromised in the supragranular layers in BA 10 and 11, relative to more posterior areas of the cortex, BA 4, 3 and 18 [36]. Based on the above study, we expected to find decreased neuron density in BA 25 in WS adults compared to TD adults, which is consistent with our results. This difference was greater in supragranular layers than in infragranular layers. In the present study, we additionally found significant increases in glia density and glia to neuron ratio, in both supragranular and infragranular layers of BA 25 in WS adults. An increase in glia was also observed in the caudate nucleus in WS, which seems to be driven by an increase in oligodendrocytes [18].

Infants: As expected, in both the TD and WS infants, overall neuron density was lower in the eight-month-old subjects than in the four-month-old subjects. In the TD infants, this difference was greatest in infragranular layers, while in WS infants there was a greater decrease in supragranular layers. Additionally, glia density increased with age in both the TD and WS infants, although this increase was far greater in WS. In the older TD infant, both neuron density and glia density were elevated compared to adult TD subjects, but glia to neuron ratio was very close to the adult mean. In the older WS infant, neuron density was elevated compared to adult WS. However, overall glia density in this subject was similar to the WS adult mean, and glia to neuron ratio was much lower.

In the youngest infant pair examined here (about four months old), differences in both neuron and glia density between TD and WS appear almost exclusively in the infragranular layers. Additionally, this was the only pair examined in which the WS subject had lower glia density than the TD subject. In the older infant pair (about eight months old) examined here, differences in neuron and glia density occurred in a similar pattern as was seen in adults. There was a greater difference in neuron density in supragranular layers, and a greater difference in glia density in infragranular layers, suggesting that this pattern is not present at birth, but is established in infancy. At both time points in the infants, overall neuron density was lower in WS than TD, as it is in adults, but the difference in neuron density in the supragranular layers may develop postnatally, in infancy. In the four-month-old infant pair, glia density was lower in WS than in TD, in contrast to all other pairs examined. Although these results represent only two WS infants, one at each age point, this suggests there may be disruptions in prenatal gliogenesis or glial migration in WS, followed by a significant increase in glial cells which begins in infancy, and continues beyond the ages of the infants included in this study.

Here, we demonstrated decreased neuron density in WS compared to TD subjects starting in early infancy; and increased in glia density in WS older infants and adults compared to TDs, but not in the youngest infant pair examined. Although the exact mechanisms for the decrease in neuron density, and the differences in glia development observed here in BA 25 in WS are unknown, they may be due in part to the deletion of *GTF2I*, *GTF2IRD1*, and *FZD9* genes crucially involved in neural development, cell division, and cell fate and neuroinflammatory processes increasing glia and decreasing neuronal survival [37,38,39]. Given that the decrease in neuron density in BA 25 is present even early in the first year of life, it likely results from a combination of deficient neurogenesis prenatally and increased apoptosis prenatally, possibly extending slightly into the postnatal period. WS neural progenitor cells (NPCs), differentiated from WS induced pluripotent stem cells (iPSCs), were found to have increased doubling time, resulting in a smaller population of NPCs, and increased levels of apoptosis compared to TD NPCs. *FZD9* appears to be critically involved in these processes in WS. By restoring *FZD9*, both apoptosis and the doubling time of WS NPCs and apoptosis were reduced to a similar level as in TD controls, creating the same number of NPCs as TD controls [37].

The increase in glia density in BA 25 in WS could be due to excess production of glial cells, deficits in apoptosis, or disrupted migration. Glia have critical roles in neural development and neurological functions, affecting neuronal survival, and synapse formation, elimination, and functioning [40,41,42]. Changes in glia cells, or in the ratio of glia to neurons, can alter the typical course of neurodevelopment and the formation and functioning of neural circuits [43]. Abnormalities in glia cells have been linked to many neurological or neurodevelopmental disorders, including major depressive disorder, ASD, and schizophrenia [42,43,44]. Decreased glia density has been found in the orbitofrontal cortex of subjects with major depressive disorder, and increased microglia density has been found in the prefrontal cortex in both ASD and schizophrenia [45,46,47].

Deletion of *FZD9* gene has been shown to affect neural progenitor cells through the canonical Wnt pathway, a pathway necessary to inhibit the differentiation of oligodendrocyte progenitor cells (OPCs) [37,38]. The proliferation of oligodendrocytes occurs in a series of successive waves, beginning prenatally, with later generated cells replacing earlier derived populations [38,48,49]. OPCs continue to proliferate while migrating to white matter until an appropriate density of OPCs has been reached [50]. OPCs remain proliferative in the subventricular zone throughout postnatal life, although cell turnover is low in typically developing adults [38,50].

In typical development, neural stem cells switch from neurogenic to gliogenic, producing astrocytes and then oligodendrocytes, in the prenatal period. The timing of this switch is critical. If the switch happens too early, it can result in overproduction of astrocytes and deficits in some neuronal populations. If it occurs too late, this can reduce the number of astrocytes produced, limiting the signals they provide for axonal guidance, neuronal survival, and synaptogenesis [43,51,52,53]. Chronic neuroinflammation, which is found in many neurological disorders, could cause increased density and activation of microglia, and potentially atrophy in astrocytes, leading to the excessive pruning of synapse and neuronal death. This may result in underconnectivity in the brain and contribute to the phenotypes of neurodevelopmental disorders [42,44,54]. Inflammation may also alter synaptic transmission through changes to astrocyte function, further affecting cognition and behavior [42].

In contrast to the findings in WS, increases in neuron number have been found in the PFC of young subjects with ASD, age range 2–16 years, with no significant difference in glia number [55]. Impaired connectivity between regions critical to social cognition and emotional regulation may be a common factor underlying some of the social and emotional abnormalities seen in both ASD and WS. In ASD, reduced fractional anisotropy was found in white matter adjacent to the vmPFC, and in the temporal lobe approaching the amygdala, suggesting disrupted connections between frontal and limbic brain regions [56]. Additionally, there is evidence of atypical activation of the vmPFC while evaluating emotional faces, as well as altered functional connectivity in fronto-striatal and fronto-amygdala circuits [57,58]. Although no differences were found in total glia number in the PFC, increased density, along with increased activation, of microglia has been demonstrated in the PFC in ASD [46]. The increased activation of microglia may reflect ongoing neuroinflammatory processes, which may contribute to loss of synaptic connections and under-connectivity in ASD [46].

Neuroinflammatory mechanisms could account for the increased glia density in WS. Although no studies have been conducted to examine microglia in WS specifically, chronic neuroinflammation is common in many neurodevelopmental disorders, including ASD and schizophrenia, and is often the cause of increased glia density [42,44,46,54]. The results from infant subjects suggest that the increased glia density in WS may develop over the first year of life, but it does not appear to be present before 4 months of age. It is not currently known if this increase is restricted to certain types of glia and to frontal and striatal regions, or if it represents a systemic perturbation of glia. Further investigation, utilizing immunohistochemical staining to determine what type of glia cells are affected, and examination of more brain regions in WS could help elucidate this matter.

The results presented here, combined with prior findings of decreased neuron density in BA 10 and 11 in WS, suggest that neuronal abnormalities in WS may be a common feature across the PFC [17]. Additionally, abnormalities have been reported in the striatum and the amygdala in WS, two subcortical structures heavily connected to BA 25 [18,20]. Together, these findings suggest that abnormalities in PFC cytoarchitecture, and altered prefrontal inhibitory control of the amygdala and striatum, may be linked to the atypical anxiety and social behaviors characteristic of WS.

## Figures and Tables

**Table 1:** Subject Information.

Subject	Age at Death	Sex	Hemisphere	PMI (h)	Cause of Death
WS 7	114 days	M	R	<30	Multiorgan failure
TD 5883	110 days	M	R	34	Sudden unexplained death in infancy
WS 2	245 days	F	R	N/A	Sudden infant death syndrome
TD 4392	234 days	F	R	13	Intussuseption of Meckel’s diverticulum
WS 10	18 years	M	R	24	Cardiac complications
TD 4916	19 years	M	R	5	Drowning
WS 15	24 years	F	R	20	Pneumonia, Sepsis
TD 5350	25 years	F	R	26	Sepsis
WS 1	31 years	M	R	26	Cardiac complications
TD 5539	31 years	M	R	24	Acute drug intoxication
WS 14	42 years	F	R	18	Cardiac complications
TD 5445	42 years	F	R	10	Pulmonary thromboembolism
WS 9	43 years	F	R	12	Cardiac complications
TD 4636	43 years	F	R	19	Pulmonary thromboembolism

WS: Williams Syndrome; TD: typically developing control; PMI: post mortem interval in hours.

**Table 2:** Mean Neuron Density (neurons/mm^3)^ and Standard Deviation in BA 25.

Cortical Layers	II/III	V/VI
**TD**	30,882 ± 2537	36,506 ± 2567
**WS**	25,594 ± 4157	33,094 ± 4417
**% Difference**	−17%	−9%

**Table 3:** Mean Glia Density (glia/mm^3)^ and Standard Deviation in BA 25.

Cortical Layers	II/III	V/VI
**TD**	18,756 ± 426	19,721 ± 465
**WS**	34,355 ± 2038	42,510 ± 1844
**% Difference**	+83%	+116%

**Table 4:** Glia to Neuron Ratio in BA 25.

Cortical Layers	II/III	V/VI
**TD**	0.61	0.54
**WS**	1.38	1.30
**% Difference**	+125%	+140%

**Table 5:** Summary table of results.

Age	Layer	Neuron Density	Glia Density
4 months	II/III	No difference	No difference
	V/VI	33% Lower	16% Lower
8 months	II/III	35% Lower	5% Higher
	V/VI	16% Lower	16% Higher
Adult	II/III	17% Lower *	83% Higher *
	V/VI	9% Lower	116% Higher *

* Statistically significant results.
